# Mouse Primordial Germ Cell-Like Cells Lack piRNAs

**DOI:** 10.1016/j.devcel.2022.11.004

**Published:** 2022-12-05

**Authors:** Navin B. Ramakrishna, Giorgia Battistoni, M. Azim Surani, Gregory J. Hannon, Eric A. Miska

**Affiliations:** 1 Wellcome/CRUK Gurdon Institute, University of Cambridge, Cambridge, CB2 1QN, UK; 2 Department of Genetics, University of Cambridge, Cambridge, CB2 3EH, UK; 3 Department of Physiology, Development and Neuroscience, University of Cambridge, Cambridge CB2 3EL, UK; 4 CRUK Cambridge Institute, Li Ka Shing Centre, University of Cambridge, Cambridge, CB2 2RE, UK; 5 Wellcome/MRC Cambridge Stem Cell Institute, University of Cambridge, Cambridge, CB2 0AW, UK; 6 Wellcome Sanger Institute, Wellcome Genome Campus, Cambridge, CB10 1SA, UK

## Abstract

PIWI-interacting RNAs (piRNAs) are small RNAs bound by PIWI-clade Argonaute proteins that function to silence transposable elements (TEs). Following mouse Primordial Germ Cell (mPGC) specification around E6.25, fetal piRNAs subsequently emerge in male gonocytes from E13.5 onwards. The *in vitro* differentiation of mPGC-Like Cells (mPGCLCs) from mESCs has raised the tantalizing prospect of studying the fetal piRNA pathway in greater depth. However, using single-cell RNA-seq and RT-qPCR along mPGCLC differentiation, we find that piRNA pathway factors are not yet expressed in D6 mPGCLCs. Moreover, we do not detect piRNAs across a panel of D6 mPGCLC lines using small RNA-seq. Our combined efforts from two laboratories highlight that *in vitro* differentiated D6 mPGCLCs do not yet resemble E13.5 or later mouse gonocytes where the piRNA pathway is active. This Matters Arising paper is in response to von Meyenn et al. (2016). See also the Correction by (Journal-to-fill-in) et al. (2022), published in this issue.

**Figure:**
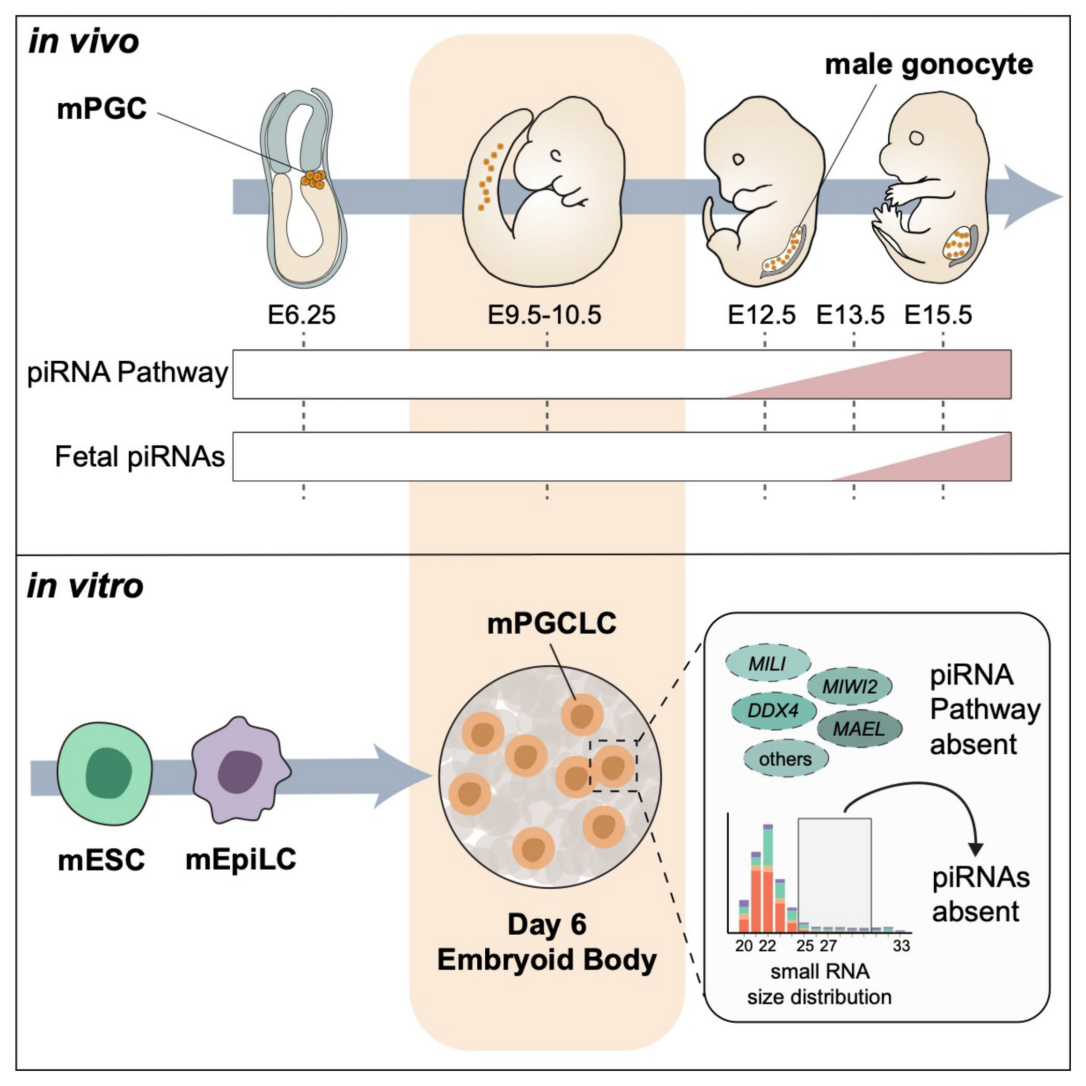

## Introduction

PIWI-interacting RNAs (piRNAs) are small non-coding RNAs with hallmarks of 2′-*O*-methyl-modified 3′ ends and a 5′-U bias. These are bound by the PIWI-clade of Argonaute proteins (PIWI proteins) (Czech et al., 2018; Özata et al., 2019; Weick and Miska, 2014). Their expression is mostly restricted to the germlines of animals, where they are frequently complementary to Transposable Elements (TEs). Knockouts of PIWI genes across metazoa cause a decline in piRNAs that results in the upregulation of TEs and, ultimately, lower fertility or sterility. This emphasizes the key roles piRNAs play as a line of defense against TE mobility, maintaining genomic integrity, and ensuring faithful transmission of genetic information (Czech et al., 2018; Özata et al., 2019; Weick and Miska, 2014).

**Figure 1:**
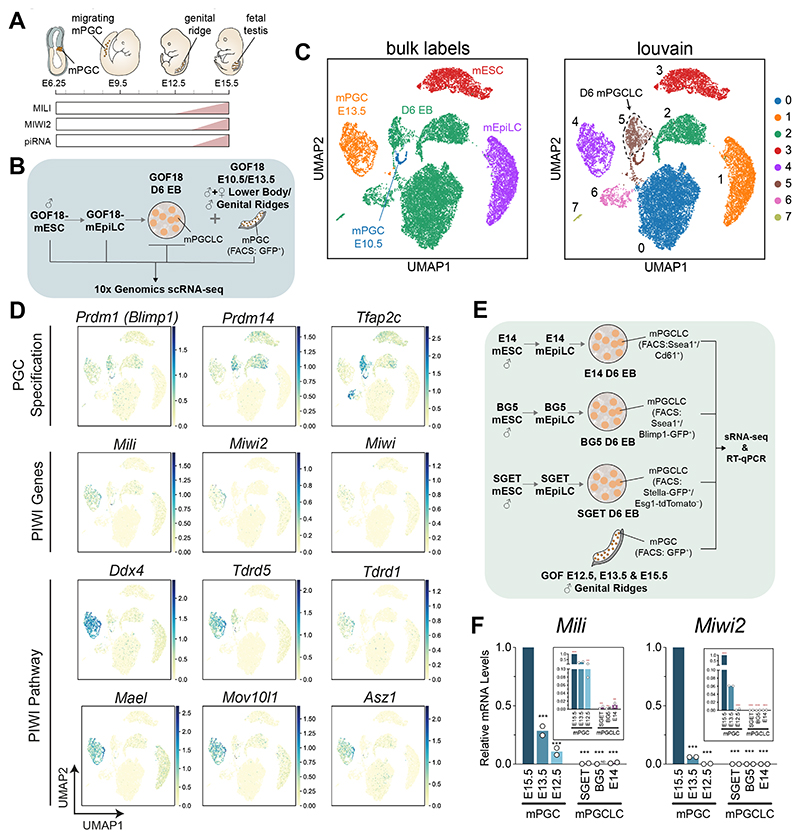
piRNA Pathway Members Absent in D6 mPGCLCs. (A) Schematic of male mPGC specification, migration and maturation into gonocytes. The expected expression of PIWI paralogs are shown, with MILI upregulated from E12.5, MIWI2 upregulated from E13.5, and with the emergence of piRNAs from E13.5. (B) Scheme of samples used in 10x Genomics single-cell RNA-seq (scRNA-seq) through the GOF18-mPGCLC induction protocol and from *in vivo* isolated E10.5 and E13.5 male mPGCs. (C) UMAP plots of scRNAseq profiles, color coded by sample of origin (replicates are merged) and membership to louvain clusters. (D) UMAP plots colored by the expression levels of key genes involved in: PGC specification, core piRNA pathway, piRNA biogenesis. Scales are logarithmic and rescaled for each gene. (E) Scheme of mPGCLC induction and isolation across three mESC lines, as well as isolation of *in vivo* isolated E12.5 and E13.5 and E15.5 male mPGCs. Long RNA fractions (>200 nt) were used in RT-qPCR experiments, while small RNA fractions (<200 nt) were used for small RNA-seq (sRNA-seq) experiments. (F) Relative *Mili* and *Miwi2* mRNA levels as detected by RT-qPCR. Normalized to *Arbp*, relative to E15.5 mPGCs (value of 1.0), with rescaled y-axis shown in inset. One-way ANOVA performed, with multiple comparisons against E15.5 mPGC (black asterisks:***p<0.001) or against E13.5 mPGC in inset (red asterisks:***p<0.001, **p<0.01, ND= not detected). n=2; all data points shown, unless not detected (ND).

In the mouse, three types of piRNAs are made in the male germline. Fetal pre-pachytene piRNAs (26 to 29-nucleotide (nt) long) are made from E13.5 gonocytes onwards (Fig 1A). These are reliant on PIWI paralogs Piwil2 (Mili) and Piwil4 (Miwi2) (Aravin et al., 2008; Aravin et al., 2009; Molaro et al., 2014). Early postnatal pre-pachytene piRNAs additionally include 3'UTR-derived piRNAs (Robine et al., 2009; Li et al., 2013). From P14 onwards, 30-nt long pachytene piRNAs bind to Mili and Piwil1 (Miwi) (Aravin et al., 2008). Knockouts of any three of these proteins result in spermatogenesis-arrest and male infertility (Carmell et al., 2007; Deng and Lin, 2002; Kuramochi-Miyagawa et al., 2004), as do knockouts of additional key members of the piRNA biogenesis pathway which process long piRNA-precursor transcripts into shorter mature piRNAs (Özata et al., 2019).

A considerable amount of genetic and biochemical work has been performed on piRNAs in the mouse, identifying key players in the biogenesis and function of piRNAs. In particular, Mili and nuclear-localized Miwi2 have been proposed to be linked to DNA methylation and H3K9me3 deposition by DNMTs and Setdb1, respectively. This occurs especially over evolutionarily-young TEs from E15.5 onwards when male mouse gonocytes undergo re-methylation from a hypomethylated state (Aravin et al., 2008; Barau et al., 2016; Kuramochi-Miyagawa et al., 2008; Liu et al., 2014; Molaro et al., 2014; Pezic et al., 2014; Yang et al., 2020). Biochemical studies have identified Tex15 and Spocd1 as direct interactors of Miwi2 (Schöpp et al., 2020; Zoch et al., 2020) and has implicated them in the afore-mentioned nuclear transcriptional silencing processes. Spocd1 was also found to bind Dnmt3a and Dnmt3l, but direct or bridging biochemical interaction to Dnmt3c or Setdb1 has thus far remained elusive (Schöpp et al., 2020). Separately, aspects of the upstream transcriptional activation of fetal piRNAs remain largely unknown, as are details on the targeting mechanisms of TEs (Goh et al., 2015; Li et al., 2013).

*In vitro* modeling of mouse Primordial Germ Cell (mPGC) specification from mouse Embryonic Stem Cells (mESCs) (Hayashi et al., 2011) has allowed for the thorough genetic and molecular dissection of mPGC specification, a process which occurs at E6.25 (Ohinata et al., 2005) (Fig 1A). The transcriptional profile, DNA methylation landscape and histone modification profiles of Day 6 (D6) mPGC-Like Cells (mPGCLCs) resembles E9.5~E10.5 mPGCs, before sex specification in mPGCs occurs (Adams and McLaren, 2002; Sabour et al., 2011, Huang et al., 2021; Kurimoto et al., 2015) and when epigenetic reprogramming of the germline remains incomplete (Hayashi et al., 2011; Ishikura et al., 2016, 2021; Hill et al., 2018; Seisenberger et al., 2012; Shirane et al., 2016; Miyoshi et al., 2016).

The advent of this *in vitro* system has created excitement about the prospect of being able to scale up biochemical and genetic studies of murine fetal piRNA biogenesis and function *in vitro*, since E15.5-like piRNA levels in a sample of D6 E14-mESC-derived mPGCLCs was previously reported (Meyenn et al., 2016).

We set out to use the *in vitro* differentiation approach to study piRNA biology. Along that path, we investigated if fetal piRNAs can be robustly identified in D6 mPGCLCs. Here, in combined, independent work from two laboratories using a total of four previously established mESC lines, we show that components of the piRNA pathway are not yet expressed in D6 mPGCLCs by single-cell RNA-seq and RT-qPCR, and that piRNAs cannot be detected in D6 mPGCLCs. Instead, we demonstrate here that mPGCLCs expectedly do not yet resemble male gonocytes, warranting the future investigation of more mature gonocyte-like cell models to achieve an *in vitro* system to study mammalian piRNAs.

## Results

### The piRNA Pathway Machinery is Not Expressed in mPGCLCs

To generate mPGCLCs *in vitro*, 2i/LIF mESCs are sequentially differentiated to germline-competent mouse Epiblast-Like Cells (mEpiLCs) followed by Embryoid Body (EB) formation in the presence of BMP, from which a proportion of cells are specified as mPGCLCs from Day 2 to 6 (Hayashi et al., 2011), which we have previously used as models with our established lines to dissect the gene regulatory and epigenetic networks of mPGC specification (Cheetham et al., 2018; Hackett et al., 2013, 2017, 2018; Murakami et al., 2016; Tischler et al., 2019). In order to examine the putative upregulation of piRNA pathway members throughout this differentiation protocol, we performed single-cell RNA-seq (scRNAseq) using the 10x Genomics platform, circumventing the need for isolation of mPGCLCs from EBs. In brief, we profiled GOF18ΔPE-EGFP (GOF18)-mESCs (FVB/C57BL/6J/129 mixed background strain, XY) (Yeom et al., 1996; Yoshimizu et al., 1999), mEpiLCs and three whole D6 EBs from the same differentiation experiment for consistency, as well as isolated *in vivo* GOF18-mPGCs (CBA/CaJ/C57BL/6J mixed background strain) (Szabó et al., 2002) collected from an E10.5 pooled litter and an E13.5 male embryo. Importantly, the latter embryonic stage acts as a positive control for when the piRNA pathway is expressed and active, with piRNAs emerging from E13.5 (Figure 1A and B). Successful mPGCLC specification was simultaneously validated by flow cytometry of D6 EBs according to the established dual staining strategy (Hayashi et al., 2011), and as similarly used by von Meyenn et al, 2016 (Figure S1A-B).

The resulting scRNA-seq transcriptome profiles were analyzed and plotted on a UMAP space (Becht et al., 2019) in order to capture both the local gene expression similarities between cells and the global structure of the dataset in regard to sample relatedness. Cells clustered according to the sample of origin (Figure 1C, left panel), and good correlation was observed for the three replicates of D6 EBs (Figure S2A). Of note, D6 EBs were composed of five different cell populations, one of which closely clustered with *in vivo* mPGCs from E10.5 embryos (Figure 1C, right panel). To further support their relatedness, both populations belonged to the same Louvain cluster 5 (Figure 1C). We additionally identified this EB-derived sub-population as mPGCLCs based on the expression of the tripartite transcription factor network for mouse germ cell specification (*Prdm1, Prdm14* and *Tfap2c*; Figure 1D), in addition to other known pluripotency and early germ cell markers (Figure S2B) (Hayashi et al., 2011). Having identified the mPGCLC sub-population, we then assessed the expression of core components of the piRNA pathway. We detected increasing expression of fetal PIWI genes *Mili* and *Miwi2* in mPGCs from E10.5 and E13.5, as well as an expectedly low expression of the adult PIWI gene *Miwi* (Figure 1D). The former two PIWI paralogs are known to be expressed incrementally and in succession in male mPGCs (Aravin et al., 2008; Figure 1A). In contrast, mPGCLCs were devoid of any expression of *Miwi* and *Miwi2*, whereas *Mili* expression levels were significantly lower than any of the *in vivo* mPGCs and similar to those detectable in mEpiLCs. Additionally, while other emergent members of the piRNA pathway were detectable in E13.5 mPGCs, they were not detected in D6 mPGCLCs at appreciable levels (Figure 1D and S2B).

To validate this observation while excluding the possibility of cell line bias, mPGCLCs were specified and dual-marker sorted from D6 EBs using three further independent mESC lines encompassing different mouse strain mixed backgrounds. This included reporter-free E14-mESCs (129/Ola strain, XY; Ssea1-positive/Cd61-positive) (Hooper et al., 1987) which was similarly used by von Meyenn et al., 2016 in their solitary small RNA library. The two other lines used were previously established in our prior work extensively characterising mPGCLCs with exquisite markers for the purposes of mPGCLC isolation. These included germline reporters Blimp1-GFP (BG5)-mESCs (C57BL/6J/DBA/129 mixed-background strain, XY; Ssea1-positive/Blimp1-GFP-positive) (Ohinata et al., 2005; Tischler et al., 2019), and Stella-GFP Esg1-tdTomato (SGET)-mESCs (B6CBA/129 mixed-background strain, XY; Stella-GFP-positive/Esg1-tdTomato-negative) (Hackett et al., 2018, Cheetham et al., 2018) (Figure 1E and S1C-H). Sorted cells were validated as *bona fide* mPGCLCs based on their expression of pluripotency and germline markers via RT-qPCR of isolated long RNA fraction (>200 nt), with their respective progenitor mESCs and mEpiLCs as controls (Figure S2C). In particular, the expected germline/pluripotency markers *Pou5f1*, Sox2, *Nanog, Zfp42* and *Klf2* were present and upregulated compared to parental mEpiLCs, while early germline-specific markers *Prdm1* (Blimp1), *Nanos3, Stella* and *Vasa* were upregulated, alongside the expected downregulation of epiblast markers *Fgf5, Dnmt3a* and *Dnmt3b*. RT-qPCR of mPGCLCs was additionally performed for the key embryonic PIWI genes in comparison with isolated male *in vivo* mPGCs from a different GOF18 mouse line (FVB/C57BL/6J mixed background strain) (Anderson et al., 1999) as positive controls (Figure 1F). The first PIWI paralog to appear in the mouse germline, *Mili*, can be readily detected from E12.5 with a marked increase through to E15.5, while *Miwi2* is detectable later from E13.5, when piRNAs emerge (Figure 1F). Importantly both transcripts were relatively absent in all three mPGCLC lines, with *Mili* and *Miwi2* mRNA levels significantly lower compared to the earliest time point of E12.5 (Figure 1F). Overall, these lines of evidence argue against the presence of piRNA pathway components gene expression in D6 mPGCLCs.

### *Bona fide* piRNAs are undetectable in D6 mPGCLCs

The absence of a robust transcriptional presence of piRNA pathway genes in D6 mPGCLCs suggests the absence of piRNA biogenesis at this stage. To confirm this, we performed small RNA-seq on the small RNA fraction (<200 nt) of the above D6 mPGCLCs, with male mPGCs acting as suitable controls.

**Figure 2:**
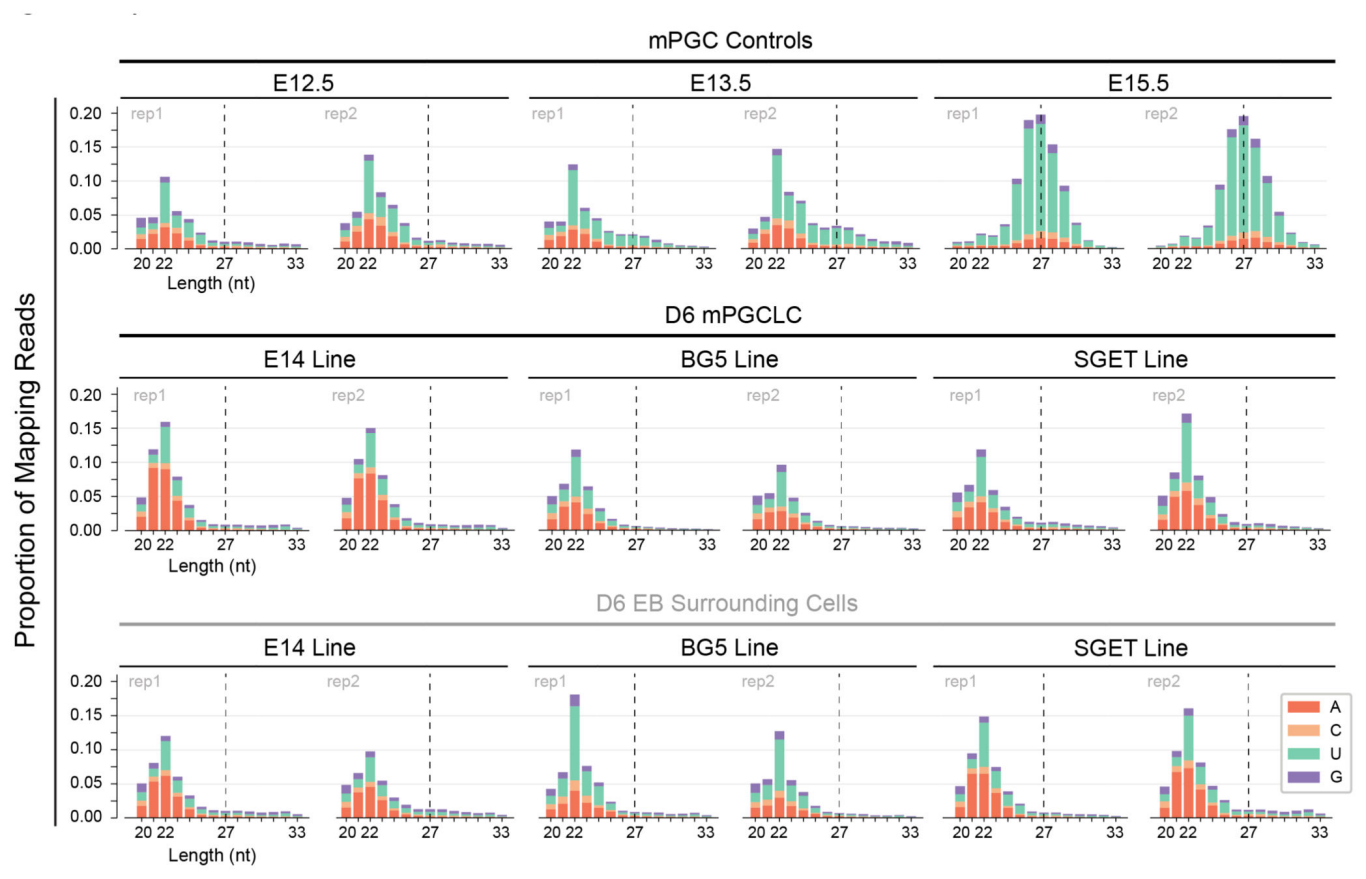
piRNAs Undetectable in Day 6 mPGCLCs. Small RNA length distributions and 5′-nucleotide bias of sorted mPGCs, various lines of sorted Day 6 (D6) mPGCLCs alongside their respective surrounding EB negative control cells. Y-axis represents the proportion of genome-mapping non-collapsed reads. Expected fetal piRNA peak of 27-nt indicated with a dashed line for clarity. n=2; all replicates shown.

Aligned small RNA reads were displayed in bar plots representing small RNA lengths and 5′ nucleotide bias (Figure 2). In the negative control of E12.5 mPGCs, no fetal pre-pachytene piRNA signature of a 27-nt 5′-U-bias peak was detected, in agreement with the absence of piRNAs at this time point (Aravin et al., 2008), with a peak profile consistent with a 22-nt miRNA peak. The E13.5 mPGC sample acted as an important control as an emergent 27-nt-peak 5′-U-bias population of piRNAs could be detected. This signature was more clearly pronounced when reads were collapsed to remove the bias of other abundant small RNA species, while emphasizing the rich sequence diversity of piRNAs (Figure S2D), demonstrating the overall sensitivity of this method. Meanwhile, the E15.5 mPGC sample acted as a robust positive control with a 6.6-fold larger 27-nt fetal piRNA peak than at E13.5. Despite the ability to detect a clear dynamic range of piRNA levels across the *in vivo* controls, no piRNA signature could be detected across all three FACS-isolated D6 mPGCLCs, with the overall small-RNA profile similar to the E12.5 mPGC negative control (Figure 2). piRNA peaks were also not detectable in mPGCLC samples when small RNA reads were collapsed (Figure S2D). Moreover, small RNA-seq of FACS-isolated non-mPGCLC EB cells as additional controls similarly showed an expected absence of piRNA signatures (Figure 2 and S2D).

## Discussion

The development of an *in vitro* model of mPGC specification (Hayashi et al., 2011) has allowed for the detailed molecular characterization of murine germline specification, with the identification of key transcription factors orchestrating this process, as well as histone modification and DNA methylation dynamics in the differentiation of mESCs through to mPGCLCs (Hackett et al., 2018; Kurimoto et al., 2015; Murakami et al., 2016; Nakaki et al., 2013; Saitou and Hayashi, 2021; Tischler et al., 2019; Zhang et al., 2018). Importantly, this standard protocol recapitulates the specification of mPGCs at E6.25 and their further differentiation up till migratory mPGCs, with Day 6 mPGCLCs sharing the same transcriptomic signature as migratory E9.5 mPGCs (Hayashi et al., 2011; Ishikura et al., 2021; Ohta et al., 2020), a stage when germ cells remain bipotential. Analysis of the DNA methylation landscape has revealed that genomic epigenetic reprogramming has commenced in mPGCLCs, but that demethylation remains incomplete and shares similar features to that of early migratory, pre-gonadal mPGCs (Ishikura et al., 2016, 2021; Meyenn et al., 2016; Shirane et al., 2016; Miyoshi et al., 2016). DNA methylation has been shown to further drop sharply only upon entry in the genital ridges up to E12.5, as sensitively detected via mass spectrometry analysis (Hill et al., 2018; Huang et al., 2021). Repressive histone modifications have been demonstrated to safeguard the demethylated genome from unruly TE expression from this timepoint onwards (Huang et al., 2021). Importantly, TE upregulation has been detected in PRC2-perturbed E13.5 mPGCs, but not in PRC2-perturbed mPGCLCs due to their incomplete demethylation (Huang et al., 2021).

Here, we show that there is no evidence of the presence of an active piRNA pathway in canonical D6 mPGCLCs, across a panel of four pluripotent mouse cells lines derived from four different mixed background strains. Our scRNA-seq results identify a population of D6 mPGCLCs with a transcriptional profile that resembles that of migratory mPGCs (E9.5~E10.5) as opposed to more mature gonadal mPGCs (E13.5 onwards). This is also further supported by the observation that D6 mPGCLCs closely cluster with our earliest time-point of E10.5 migratory mPGCs and are well separated from the population of E13.5 male gonocytes (Figure 1C). The expression of the core members of the piRNA pathway follows the same pattern with *Mili, Miwi2*, and other crucial piRNA biogenesis factors being detectable in E13.5 mPGCs yet absent in both mPGCLCs and E10.5 mPGCs, in agreement with previous work in mPGCLCs (Ishikura et al., 2021; Zhao et al., 2021). Indeed, the expression of genes associated with the piRNA pathway has been demonstrated to be strongly upregulated upon DNA demethylation in mPGCs (Hackett et al., 2012), a process not yet complete in mPGCLCs in the absence of further induced differentiation (Hackett et al., 2012; Ishikura et al., 2021; Ohta et al., 2020; Saitou and Hayashi, 2021). As expected, based on our characterization of piRNA pathway gene expression in mPGCLCs by scRNAseq and qPCR, no piRNAs could be detected in D6 mPGCLCs across a panel of three different mouse pluripotent cell lines, despite sensitive detection of piRNAs in E13.5 mPGCs onwards via small RNA-seq.

Our observations are incompatible with a previous report that piRNAs are abundant in a single replicate of E14-mESC-derived mPGCLCs, which showed piRNA levels similar to that of their single replicate of mature GOF18 E15.5 male gonocyte control (Meyenn et al., 2016). A Correction is now published on the original paper to confirm that the data from the analysis of their small RNA-seq mPGCLC library was incorrect.

Why mPGCLCs seemingly stall in their maturation in EBs has been a matter of keen interest (Cooke and Moris, 2021; Saitou and Hayashi, 2021). Further *in vitro* maturation of male mPGCLCs to gonocyte-like cells, and even further through spermatogenesis to adult spermatid-like cells, or of female mPGCLCs to oocyte-like cells, tend to involve the isolation of mPGCLCs from EBs and reaggregation with either male or female gonadal somatic cells respectively (Hikabe et al., 2016; Ishikura et al., 2021). The surrounding gonadal somatic cells have been postulated to provide the correct cellular architecture and signaling niche for sex-specific maturation of germ cells that the EB environment lacks - recapitulating the entry of migratory mPGCs to the genital ridges from E10.5 onwards (Adams and McLaren, 2002; Cooke and Moris, 2021). In male mPGCs, this is coupled with (i) further DNA demethylation, (ii) activation of the piRNA pathway with upregulation of piRNAs from E13.5, (iii) mitotic-arrest from E14.5, and (iv) DNA remethylation largely complete by E16.5 (Hill et al., 2018; McLaren, 1984; Ramakrishna et al., 2021; Zhao et al., 2021). *In vitro* recapitulation respecting the sequential order of the above four processes should be pursued as it bears crucial implications for understanding the epigenetic integrity of the resulting *in vitro* derived gametes and, potentially, for the physiology of the resulting offspring. In parallel, an *in vitro* model will be useful for scalable biochemical dissection of the mammalian fetal pre-pachytene piRNA pathway, akin to the OSC cell line which acts as a model for *Drosophila* primary piRNAs (Saito et al., 2009, 2010). To this end, it will be of interest to assay the presence of fetal piRNAs in more mature gonocyte-like cells derived in a recently published protocol for the full differentiation of *in vitro* derived gametes (Ishikura et al., 2021), where the authors show transcriptional activation of genes of the fetal pre-pachytene piRNA pathway upon co-culture of expanded mPGCLCs with dissociated E12.5 male gonadal somatic cells.

## Methods

### Animals

Animal studies were authorized by UK Home Office Project Licenses (PE596D1FE, 70/8412) and carried out in Home Office-designated facilities in the Wellcome/CRUK Gurdon Institute and CRUK Cambridge Institute. Two different GOF18ΔPE-EGFP (GOF18) mouse strains were used as specified in the text: TgOG2 MGI:3057158 (Szabó et al., 2002) which has a CBA/CaJ/C57BL/6J mixed background, and Tg(Pou5f1-GFP)1Scho MGI:3693125 (Anderson et al., 1999) which has an FVB/C57BL/6J mixed background. Embryo samples were taken from ethically euthanized pregnant females following timed matings at E10.5 and E13.5 from the former strain, and E12.5, E13.5 and E15.5 from the latter strain.

Embryo samples of E12.5, E13.5 and E15.5 were dissected and microdissected to isolate the genital ridges from the developing mesonephros. In the case of E10.5 embryos, the posterior part of the body below the primordial heart was collected. Tail clippings of mouse samples were placed into 50 μl of QuickExtract DNA Extraction Buffer (Lucigen), with 6 min incubation at 65°C in a heatblock, followed by 2 min at 98°C to extract gDNA. Genotyping PCR and DNA electrophoresis gel run was then performed as previously described (Tang et al., 2015), using sex genotyping primers for SRY and ZFX/Y.

### Isolation of mPGCs

Male genital ridges were digested post-genotyping (alongside confirmatory visual sex-determination for samples ≥E13.5). For RNA extraction, 2 embryos were pooled to comprise one biological replicate, with each replicate from different litters, and digested with Trypsin-EDTA for 20 min at 37°C. Cells were diluted in FACS buffer (3% FBS, 5 mM EDTA in PBS). Cells were pelleted, resuspended and sorted with Sony Cell Sorter SH800Z for 5,000 EGFP-positive cells (mPGCs) directly into Qiazol (Qiagen) and frozen at -70°C until RNA extraction. For single cell RNA-seq, samples were collected from one single embryo at E13.5, whereas one full litter was combined at E10.5. Digestion was performed with 0.3 mg/mL Collagenase-IV (Roche), 0.16% Trypsin-EDTA (LifeTechnologies) in D-PBS, at 37ºC with shaking (800 rpm) for 10 minutes for E10.5 embryos and 8 minutes for dissected E13.5 genital ridges. Digestion was blocked by adding 1 volume of DMEM+10% FBS (Life Technologies). A live cell-dead staining was also performed by incubating the cell suspension with 200 ng/mL Cell Trace Calcein Red-Orange (LifeTechnologies) and 1 mg/mL DAPI for 30 minutes. After three washes in 1 mL FACS medium II (D-PBS+2%FBS), the cell suspension was filtered through a 100 μm mesh and live mPGCs (Cell Trace positive, DAPI negative, EGFP positive) sorted with an AriaIIU Sorter (BD) directly in 28 μL cold D-PBS+0.04% BSA, and used immediately loaded in a 10x Genomics 3′ scRNAseq Chromium v2 chip.

### mPGCLC Differentiation

The standard protocol as described in Hayashi et al., 2011 was followed. Stella-GFP/Egs1-tdTomato (SGET) (Hackett et al., 2018), Blimp1-GFP (BG5) (Tischler et al., 2019), E14 (Hooper et al., 1987) and GOF18 (Yeom et al., 1996; Yoshimizu et al., 1999) XY mESC lines were used in this study. Naïve-mESCs from the SGET, BG5 and E14 lines were maintained on N2B27 medium supplemented with 2i (PD0325901 (1 μM) and CHIR99021 (3 μM; both Stemgent) and 1000 U/ml LIF (Cambridge Centre for Stem Cell Research) (2i/L) on gelatin-coated plates. GOF18 naïve-mESCs were maintained on a confluent monolayer of irradiated Mouse Embryonic Fibroblasts (MEFs, Life Technologies) on gelatin in Dulbeccos’s Modified Eagle Medium KnockOut (Life Technologies), supplemented with 15% KnockOut Serum Replacement (Life Technologies), 0.1 mM ß-mercaptoethanol (Gibco, Life Technologies), 1000 U/mL LIF (EMD Millipore), 1 mM PD03259010 (Miltenyi Biotechnologies), and 3 mM CHIR99021 (Miltenyi Biotechnologies). Prior to differentiation, GOF18-mESCs were moved to feeder free conditions for 3-4 passages on N2B27 medium supplemented with 0.1 mM ß-mercaptoethanol (Gibco, Life Technologies), 1000 U/mL LIF (EMD Millipore), 1 mM PD03259010 (Miltenyi Biotechnologies), and 3 mM CHIR99021 (Miltenyi Biotechnologies) on freshly coated laminin-ornithine plates.

For mEpiLC induction (40h) (Hackett et al., 2018; Cheetham et al., 2018), 2iL-mESC were washed with PBS and seeded onto fibronectin (16.7 mg/ml) coated plates with EpiLC medium (N2B27, 1% KSR, bFGF (12 ng/ml), Activin A (20 ng/ml)). Media was changed every day. mEpiLC were then gently dissociated and seeded at 2,000-3,000 cells per EB in ultra low-cell binding U-bottom 96-well plates (NUNC) with mPGCLC induction medium (GK15: GMEM, 15% KSR, 0.1mM NEAA, 1 mM sodium pyruvate, 0.1 mM β -mercaptoethanol, 100 U/ml penicillin, 0.1 mg/ ml streptomycin and 2 mM L-glutamine; supplemented with cytokines: BMP4 (500 ng/ml), LIF (1000 U/ml), SCF (100 ng/ml), BMP8a/b (500 ng/ml) and EGF (50 ng/ ml)). After 6 days, EBs were collected for FACS collection or scRNAseq. For FACS preparation for E14, BG5 and SGET lines, EBs were dissociated as previously described using Trypsin-EDTA for 10 minutes at 37°C, diluted in FACS medium, then pelleted and resuspended in FACS medium with antibodies Ssea1-AF660 and/or Cd61-PE as specified in the text. For FACS collection of these lines, the above-mentioned antibodies and/or respective reporters as specified in the text and Figure S2 were used for mPGCLC and surrounding EB cells sorting (5,000 cells/tube) with Sony Cell Sorter SH800Z directly into Qiazol. mESC, mEpiLC and EB live samples were imaged under a fluorescence microscope using brightfield imaging, and respective fluorescent channels.

For scRNA-seq of the GOF18 line, 2iL-mESCs and mEpiLCs were dissociated using Accutase (Millipore) and TripLE (Gibco, LifeTechnologies), respectively, and washed three times in FACS Medium II. For EBs, a gentle dissociation protocol was optimized to minimize cell loss and to avoid any potential survival bias through the pipeline. Individual D6 EBs were collected in D-PBS and dissociated in 0.05% Trypsin (Gibco, LifeTechnologies) in D-PBS for 8 minutes at 37°C. Digestion was blocked by adding 1 volume of DMEM+10% FBS (Life Technologies). Cells were washed 3 times in FACS Medium II and manually counted on a Neubauer chamber before loading on a 10x Genomics 3′ scRNAseq v2 Chromium chip (target 7,000 cells/channel). In parallel, FACS validation of mPGCLC differentiation of the EBs was performed on AriaIIUSorter (BD) using the EGFP reporter, and antibodies Ssea1-AF647 and Cd61-BV421.

### Small RNA-seq

Using Qiazol cell lysates, the column-based miRNeasy-micro kit (Qiagen) was used to extract both <200 nt (small RNA) and >200 nt (long RNA) fractions from the same sample, according to the manufacturer's instructions. Small RNA library preparation was done following the instructions for lowest input of the NextFlex Small RNA-Seq Kit v3 for Illumina (Perkin Elmer/BIoo Scientific). In particular, a prolonged overnight 16°C 3' adapter ligation step was done to enrich for 2'-O-methyl-modified 3'-end small RNAs, with the adapters having 4N random ends to reduce ligation bias. Quarter-dilutions of all adapter concentrations were used. The gel-method was used post-PCR amplification for excision of the 150-165 bp band. Libraries were quantified using Qubit and TapeStation HSD1000 reagents and multiplexed. Samples were run on both Illumina HiSeq1500 and NovaSeq 6000 platforms.

### RT-qPCR

For long RNA RT-qPCR, cDNA was synthesized using the QuantiTect Reverse Transcription Kit (Qiagen). qRT-PCR was performed using SYBR Green JumpStart Taq ReadyMix (Sigma Aldrich), performed on a QuantStudio 12K Flex Real-Time PCR machine (Applied Biosystems) in 384-well plates and analyzed using the ΔΔCT method as described previously (Irie et al., 2015). All mouse qPCR primers used are detailed in Suppl Table 1.

### Small RNA-seq Analysis

Fastq files of the same library were merged and reads were trimmed using cutadapt (v3.2) with the following parameters: -a TGGAATTCTCGGGTGCCAAGG --minimum-length 23 (thresholding reads to a minimum of 15 nt), then followed by -u 4 -u -4 to remove the 4 random NNNN adaptor ends. To identify piRNA signatures from all small RNA datasets, trimmed reads passing quality control were mapped to the GRCm38 (mm10) mouse genome allowing for up to 100 multi-mappers, and single random-assignment of multimappers, and a stringent mismatch of 2, using STAR (v2.5.3a) with the following parameters:

-- outFilterMultimapNmax 100
-- winAnchorMultimapNmax 100
-- outFilterMismatchNmax 2
-- alignIntronMax 1
-- alignEndsType EndToEnd
-- readFilesCommand gunzip -c
-- scoreDelOpen -10000
-- scoreInsOpen -10000
-- outSAMtype BAM SortedByCoordinate
-- outSAMmultNmax 1
-- outMultimapperOrder Random

An in-house custom python (v3.5.3) script (https://gitlab.com/tdido/tstk/-/blob/master/tstk/peterplot.py) was then used to generate collapsed fasta files from the BAM outputs, and to generate either collapsed or uncollapsed small RNA length distribution histograms with the 5′-nucleotide frequency displayed, normalized to all mapping reads.

### scRNAseq Analysis

All the samples were processed using the Single Cell 3′ Reagent Kit - v2 Chemistry from 10x Genomics, following the manufacturer’s instructions. GEMs were generated immediately after sorting. The final libraries, each with a unique index, were multiplexed and sequenced on an Illumina HiSeq 4000 with a target read depth of ~50,000 reads/captured cell. The raw sequencing data was parsed and mapped on the reference mm10 mouse transcription with the CellRanger software. Anndata, Numpy (Harris et al., 2020), Scanpy (Wolf et al., 2018) and Python (v3) were used for downstream analysis. Cells were filtered based on the fraction of mitochondrial transcripts (<0.2) to exclude apoptotic cells and potential background RNA, and total UMIs (<50,000/cell) to remove potential doublets. After filtering, cells were combined in a cell experiment object using the Anndata module, retaining the information about the sample of origin and the individual replicates. The aggregated data object was normalized for the library size of each cell to a total of 10,000 counts/cell, and normalized counts were converted to a natural logarithmic scale. Highly variable genes were identified (minimum dispersion = 0.5, mean expression between 0.0125 and 3) and used for dimensionality reduction and clustering. The data was regressed for the effects of the number of UMI/cell and the gene expression values were re-scaled to unit variance across all the different cells of the aggregated dataset. For the UMAP, we ordered the principal components and chose the top 12 principal components that could explain most of the variance of the dataset. Clustering was performed using the louvain approach as described in Levine et al. (2015), and implemented in Traag et al., 2019.

## Supplementary Material

Supplementary Materials

## Data Availability

Script for making small RNA length distribution plots can be found here (https://gitlab.com/tdido/tstk/-/blob/master/tstk/peterplot.py). Our GEO Accessions are: GSE197334 (token: shyfiaeoxrqjpmz) - https://www.ncbi.nlm.nih.gov/geo/query/acc.cgi?acc=GSE197334 GSE 197335 (token: kjwhsyqgplczdap) - https://www.ncbi.nlm.nih.gov/geo/query/acc.cgi?acc=GSE197335 GSE196934 (token: kjkjsuqatrybrmx) - https://www.ncbi.nlm.nih.gov/geo/query/acc.cgi?acc=GSE196934 GSE196851 (token: ktiteimwzfibdsl) - https://www.ncbi.nlm.nih.gov/geo/query/acc.cgi?acc=GSE196851
