# Supplementary material for: Mouse Primordial Germ Cell-Like Cells Lack piRNAs
Source: Dev Cell. Author manuscript; Available in PMC 2025 Sep 8. (PMC7618082; doi:10.1016/j.devcel.2022.11.004)
Supplement: Supplementary Materials [file EMS208394-supplement-Supplementary_Materials.pdf]

SUPPLEMENTARY FIGURES AND LEGENDS

Figure S1

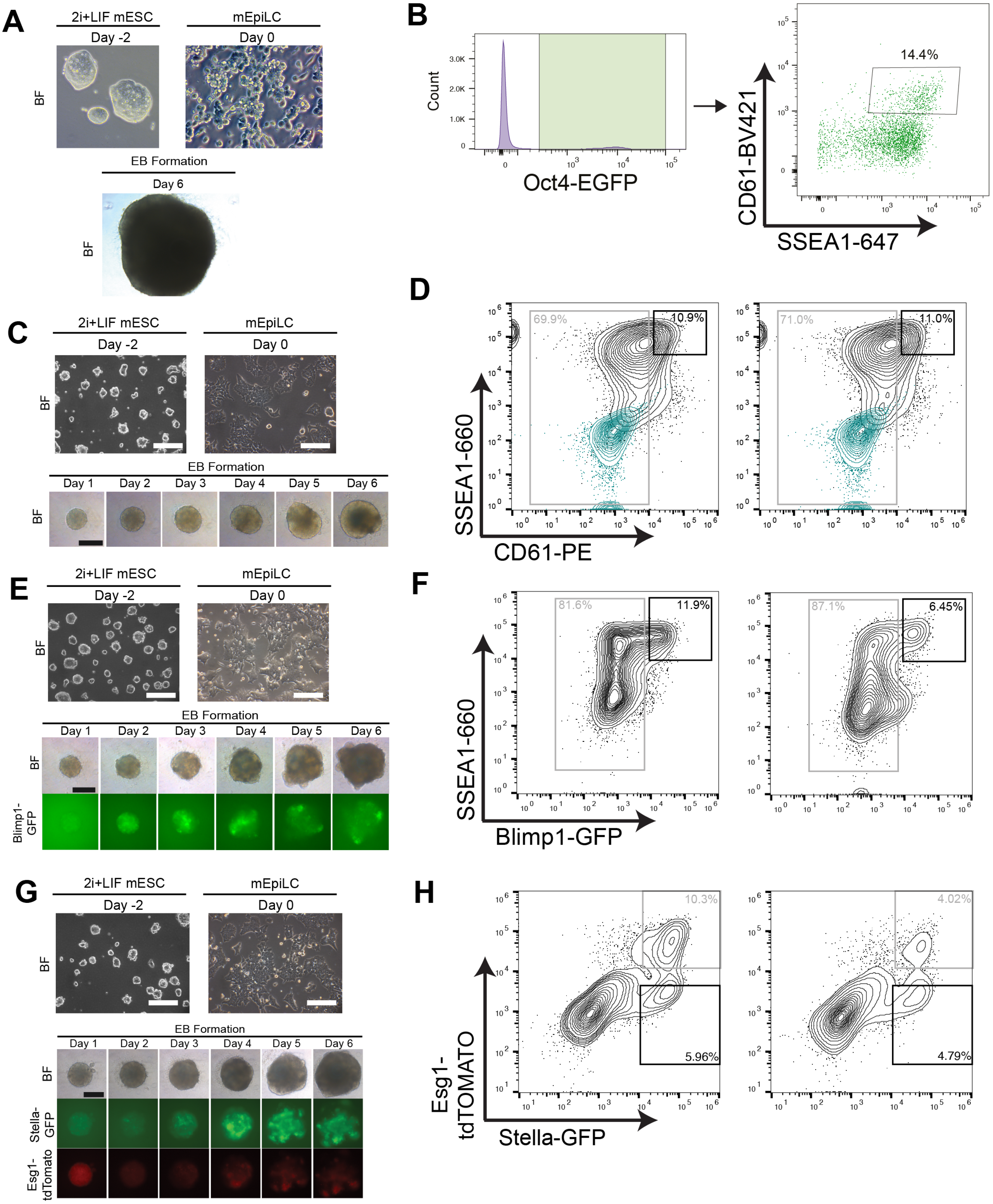

Figure S1. ***In Vitro*** **Sample Differentiation.** (A) Representative brightfield microscopy images of GOF18 mESC, mEpiLC and Day 6 Embryoid Body (EB) used in scRNA-seq experiments. GOF18 mESC and mEpiLC imaged at 4x higher magnification than the EB. (B) FACS plots validating the presence of OCT4-EGFP, SSEA1 and CD61 triple positive mPGCLCs in whole EBs used in the scRNA-seq experiment. (C,E,G) Respective microscopy images of 2i/LIF mESC, mEpiLC and Embryoid Body (EB) Day 1 to 6 for E14, BG5 and SGET cell lines. White scale bars denote 100 μm and black scale bars denote 200 μm. (D,F,H) FACS plots showing gated collection of Day 6 mPGCLC populations for E14, BG5 and SGET cell lines respectively (black box), alongside sorted negative control surrounding EB cells (grey box) using fluorescent germline reporters and/or germline cell surface markers as indicated. Negative control unstained EBs (teal) are displayed for the E14 cell line (D).

Figure S2

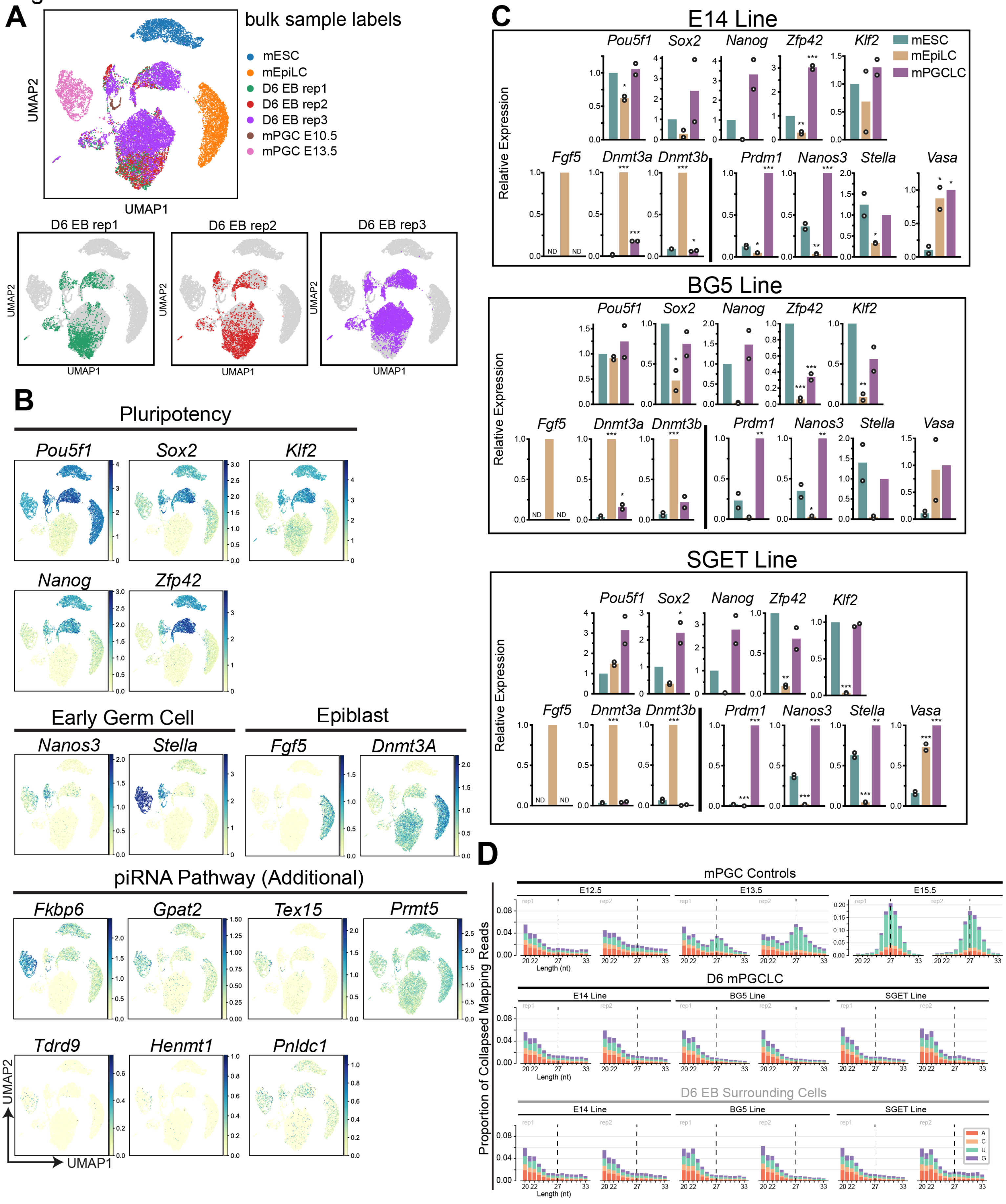
A
bulk sample labels
mESC
mEpiLC
D6 EB rep1
D6 EB rep2
D6 EB rep3
mPGC E10.5
mPGC E13.5
UMAP1
UMAP2
D6 EB rep1
D6 EB rep2
D6 EB rep3
B
Pluripotency
Pou5f1
Sox2
Klf2
Nanog
Zfp42
Early Germ Cell
Nanos3
Stella
Epiblast
Fgf5
Dnmt3A
piRNA Pathway (Additional)
Fkbp6
Gpat2
Tex15
Prmt5
Tdrd9
Henmt1
Pnldc1
C
E14 Line
BG5 Line
SGET Line
Relative Expression
Pou5f1
Sox2
Nanog
Zfp42
Klf2
Fgf5
Dnmt3a
Dnmt3b
Prdm1
Nanos3
Stella
Vasa
mESC
mEpiLC
mPGCLC
ND
D
mPGC Controls
E12.5
E13.5
E15.5
D6 mPGCLC
E14 Line
BG5 Line
SGET Line
D6 EB Surrounding Cells
Proportion of Collapsed Mapping Reads
Length (nt)
rep1
rep2
A
C
U
G

Figure S2. Validation of mPGCLCs. (A) UMAP plot of scRNAseq profiles, color coded by sample of origin, with Embryoid Body (EB) replicates indicated. Individual UMAP plots for each EB replicate shown, with all other samples greyed out. (B) UMAP plots colored by the expression levels of key genes involved in: pluripotency network, early germ cell markers, epiblast markers, and additional piRNA biogenesis members. Scales are logarithmic and rescaled for each gene. (C) Relative mRNA expression levels as detected by RT-qPCR for pluripotency markers (top), mEpiLC markers (bottom-left) and germ cell markers (bottom-right). Normalized to *Arbp*, relative to either mESC (top), mEpiLC (bottom-left) or mPGCLC (bottom-right) for E14, BG5 and SGET lines. n=2; all data points shown. One-way ANOVA performed, with multiple-comparisons against mESC. ***$p<0.001$, **$p<0.01$, *$p<0.05$, ND= not detected. (D) Small RNA length distributions and 5′-nucleotide bias of sorted mPGCs and various lines of sorted Day 6 (D6) mPGCLCs alongside their respective surrounding EB negative control cells. Y-axis represents the proportion of genome-mapping collapsed reads. Expected fetal piRNA peak of 27-nt indicated with a dashed line for clarity. n=2; all replicates shown.

SUPPLEMENTARY TABLES

Supplementary Table 1: List of qPCR Primers

Gene	Forward	Reverse
ARBP	CAAAGCTGAAGCAAAGGAAGAG	AATTAAGCAGGCTGACTTGGTTG
MIWI2	AACCGGTGGTACAGACACAA	CGAGCCGCACTCTGTTACAC
MILI	TCCAGGAGAGAGCGAGAGAG	CCAATTCCTTATCTTTCCACCACG
POU5F1 (Oct4)	CCAATCAGCTTGGGCTAGAG	CTGGGAAAGGTGTCCCTGTA
SOX2	ACCAGCTCGCAGACCTACAT	TGGAGTGGGAGGAAGAGGTA
NANOG	ACCTGAGCTATAAGCAGGTTAAGAC	GTGCTGAGCCCTTCTGAATCAGAC
ZFP42	GGCCTCTTTTGGTATTCCATGG	CCCATCCCCTTCAATAGCACAT
KLF2	TCGAGGCTAGATGCCTTGTGA	AAACGAAGCAGGCGGCAGA
FGF5	AAAGTCAATGGCTCCCACGAA	CTTCAGTCTGTACTTCACT
DNMT3A	GACTCGCGTGCAATAACCTTAG	GGTCACTTTCCCTCACTCTGG
DNMT3B	CCCATGCAATGATCTCTCTAAC	AGAATGGACGGTTGTCGC
PRDM1 (Blimp1)	GGGAAACCCAAGAGCCTTAC	GCTTGCTAGCATGTGTGGAA
NANOS3	CACTACGGCCTAGGAGCTTGG	TGATCGCTGACAAGACTGTGG
DPPA3 (Stella)	AGGCTCGAAGGAAATGAGTTTG	TCCTAATTCTTCCCGATTTTCG
DDX4 (Vasa)	CAGTTTGCAATGTGAGCTTTG	GGGGGAAATGTGTTTCATCTT

Supplementary Table 2: Small RNA Libraries

Library	Total Cleaned Reads	Total Mapping Reads	Percentage Mapping Reads (%)
mPGC_E12.5_1	12395543	7447873	60.1
mPGC_E12.5_2	21445408	13614516	63.5
mPGC_E13.5_1	19232777	12043363	62.6
mPGC_E13.5_2	31039306	19734948	63.6
mPGC_E15.5_1	19044068	13601492	71.4
mPGC_E15.5_2	36877398	26552524	72.0
mPGCLC_E14_1	12264055	8274092	67.5
mPGCLC_E14_2	12158964	8046414	66.2
mPGCLC_BG5_1	12131668	8251090	68.0
mPGCLC_BG5_2	16446437	10227279	62.2
mPGCLC_SGET_1	18697271	10209580	54.6
mPGCLC_SGET_2	15190410	9526338	62.7
Non-PGCLC_E14_1	12287433	7421061	60.4
Non-PGCLC _E14_2	20141629	12899662	64.0
Non-PGCLC _BG5_1	15122499	9453155	62.5
Non-PGCLC _BG5_2	23131777	14444567	62.4
Non-PGCLC _SGET_1	22435538	14404300	64.2
Non-PGCLC _SGET_2	16469987	9775392	59.4

Supplementary Table 3: List of Antibodies

Antibody	Conjugation	Company
Ssea1	Alexa Fluor 660	eBioscience
Ssea1	Alexa Fluor 647	BioLegend
Cd61	PE	BioLegend
Cd61	BV421	BD Biosciences

Supplementary Table 4: Percent Contribution of Bulk Samples to scRNA-seq Louvain Clusters

	louvain 0	louvain 1	louvain 2	louvain 3	louvain 4	louvain 5	louvain 6	louvain 7	Total cells
**E10.5 mPGC**	0.00%	0.00%	0.00%	0.00%	0.00%	100.00%	0.00%	0.00%	149
**E13.5 mPGC**	0.00%	0.00%	0.00%	0.00%	98.66%	1.34%	0.00%	0.00%	1417
**EBd6 Rep1**	73.37%	0.18%	13.67%	0.00%	0.00%	6.90%	5.13%	0.75%	2261
**EBd6 Rep2**	74.56%	0.09%	13.66%	0.00%	0.00%	7.77%	3.44%	0.47%	2123
**EBd6 Rep3**	67.73%	0.05%	22.41%	0.02%	0.00%	6.42%	2.96%	0.42%	6662
**mESCs**	0.00%	0.00%	0.00%	100.00%	0.00%	0.00%	0.00%	0.00%	2044
**mEpiLC**	0.03%	99.87%	0.06%	0.00%	0.00%	0.03%	0.00%	0.00%	3087
